# Characterization and kinetics analysis of the thermal decomposition of the humic substance from hazelnut husk

**DOI:** 10.3906/kim-2004-62

**Published:** 2020-12-16

**Authors:** Fatma KARA, Duygu ADIGÜZEL, Ufuk ATMACA, Murat ÇELİK, Jale NAKTİYOK

**Affiliations:** 1 Department of Chemical Engineering, Engineering Faculty, Atatürk University, Erzurum Turkey; 2 Oltu Vocational School, Atatürk University, Oltu, Erzurum Turkey; 3 Department of Chemical, Faculty of Science, Atatürk University, Erzurum Turkey

**Keywords:** Hazelnut husk, humic substance, thermal decomposition, TG-DTG/DSC analysis, kinetic analysis

## Abstract

A humic substance was obtained from hazelnut husk using an alkali extraction. The chemical and morphological structure of the humic matter was characterized via elemental analysis, Fourier transform infrared spectrometry (FTIR), nuclear magnetic resonance, Brunauer-Emmet-Teller (BET) analysis, scanning electron microscopy (SEM), and thermogravimetric-FTIR (TG-FTIR). In addition, thermal analysis measurements TG analysis-differential thermogravimetry/differential scanning calorimetry (TGA-DTG/DSC) were performed under dynamic air conditions to better determine the origin, physical and chemical structure, and decomposition process of the humic matter. The Kissinger-Akahira-Sunose (KAS) and Flynn-Wall-Ozawa (FWO) methods were used to calculate the kinetic parameters of the high-temperature decomposition process. It was observed that the activation energy values were almost constant at certain conversion and temperature intervals. In addition, the structure of the humic substance at different temperatures was also investigated via FTIR analysis. It was found that the obtained humic substance had a very stable structure and decomposed at a high temperature. The stability of the humic matter can be a useful tool in the environmental quality research of soil.

## 1. Introduction

Humic substances are one of the most important components of soil. They form a large part of the organic matter in the soil and play an effective role in its producibility. Humic substances have a macromolecular and complex structure. They consist of the chemical and biological degradation of animal and plant residues in soil, water, and sediments, so they can be obtained from any source of organic matter [1,2].

Many studies have been performed to attain humic substances from biological wastes [3–7]. They can exist in different ambients; for example soils, natural waters, rivers, lakes, sea sediment plants, and composts, but it has been observed that the chemical properties, quantities, and proportions of humic substances obtained from wastes can vary according to their sources [8–10]. There is no consensus on the structural and chemical properties of the final product to be obtained from humic substances [11].

Apart from agricultural fields, there are many different uses for humic substances, such as a dispersant in ceramic suspensions, wastewater treatment as an adsorbent, drilling fluids, medicine and creams for the treatment of various diseases, lead acid batteries as surfactant material, etc. [12]. However, they do not have certain or identified structures and features, depending on their sources and extraction conditions. Although they are chemically heterogeneous compounds with different proportions and configurations of functional groups, they comprise elements, especially C, H, N, O, and S, and the carboxyl (-COOH), amine (-NH _2_ ), hydroxyl (-OH), and phenol (Ar-OH) functional groups [13].

Alkali humates are obtained from extraction processes with alkaline solutions (NaOH, KOH solutions, etc.) of humic substance sources. They are known as water-soluble salts of humic acid and have many benefits, such as expediting plant growth/development and enhancing plant resistance against unsuitable ambient situations. Moreover, it has been stated that the preoxidation step increases the extraction efficiency of humic acid [14,15].

Thermal analysis [TG analysis-differential thermogravimetry/differential scanning calorimetry (TG/DTG-DSC)] is often utilized to describe the thermal stability or behavior of humic substances under various conditions and to investigate the kinetics of the physicochemical processes during decomposition [16–20]. It is performed for humic substances both to measure its moisture and ash contents and to monitor and characterize structural changes during the heating process. In addition, TG analysis also provides the opportunity to compare humic substances obtained from different origins. However, because of the molecular complexity of humic substances, the explication of thermal curves and related processes is not provided only by thermal analysis [21].

The kinetics descriptions of the physical and chemical phenomena formed by the decomposition through TG-DTG analysis can be determined. Kinetic parameters can be calculated by isoconversional methods (model-fitting and model free).

Model-free methods, such as Kissinger-Akahira-Sunose (KAS) and Flynn-Wall-Ozawa (FWO), are among the most popular techniques used for calculating the effective activation energy versus the conversion degree of any chemical reaction [22–27]. These methods permit the calculation of the activation energies versus conversion values without detecting the reaction mechanism [27].

(1)KAS method: lnβT2=ln[AREa]-EaRT+lndf(a)da(2)FWO method: ln(β)=ln(AEag(a)R)-5.331-1.052(EaRT)

Here, β,Ea, R, A, f(α), and g(α) are heating rate, activation energy, gas constant, preexponential factor, reaction mechanism, and integral function of f(α), respectively.

There are very few studies in the literature about the thermal decomposition of humic substances under air conditions in detail. The humic substance (potassium humate) from the hazelnut husks extracted with KOH solution will be mentioned after the oxidation step. In this study, the obtained product was examined using many analytical methods [elemental analysis, proton nuclear magnetic resonance (1H NMR), scanning electron microscopy (SEM), Brunauer-Emmet-Teller (BET) analysis, and Fourier transform infrared spectrometry (FTIR)]. The thermal behavior, stability, and decomposition kinetics of the product were determined via thermal analysis, and the evolved gases through the decomposition of the humic substance were analyzed by TG-FTIR at increments of 50 °C.

## 2. Materials and methods

The hazelnut husks used in the study were obtained from Giresun, Turkey. Hazelnut husk covers the nut shell and it is initially green, then turns yellow to red, and finally brown. During the harvest, the husks are removed from the nuts by machines. For the experiments, the samples were washed, oven-dried at 80 °C overnight, and sieved in the laboratory, all of which were used in the extraction process. As can be seen in Table 1, the ultimate analysis of the hazelnut husks comprised carbon (42.62%), hydrogen (5.2%), nitrogen (0.9%), sulfur (0.08%), oxygen (45.4% as the difference), and ash (5.8%) contents.

**Table 1:** Elemental analysis of the humic substance from the hazelnut husks.

Materials	C (%)	H (%)	N (%)	S (%)	O (%)	Ash	H/C	O/C
Hazelnut husk	42.62	5.2	0.9	0.08	45.4	5.8	1.47	0.80
Humic substance	27.6	3.04	1.40	0.05	32.61	35.3	1.32	0.89

H/C: (%H/1.00)/(%C/12.02) O/C: (%O/16.00)/(%C/12.01)

### 2.1. Extraction of humic substances

The alkali extraction process was chosen after the oxidation step with HNO3 solution to obtain the humic substance from the hazelnut husks. Next, the hazelnut husks were treated with KOH to dissolve the humic acid. Extraction with KOH solution was selected due to the properties of the potassium; for example, it is a basic nutrient for plants and improves the soil structure [28].

As a next step, 20 g of hazelnut husk was added 200 mL of 3 M HNO3. The hazelnut husk/HNO3 mixture was shaken for 3 h at 80 °C, and then filtrated. After the oxidation step with HNO3, the dried and weighed residual hazelnut husk was placed into a flask. Next,2 M KOH solution was added. It was shaken for 7 h at 80 °C. The solution, which contained soluble humic substances, was separated from the residue via filtration. The procedure was repeated 2 times and the extracts were gathered together. The extract (potassium humate solution) was completely evaporated at 60 °C. Thus, it led to the collapse of the potassium humate-components. The product, the humic substance, was dried and weighed. It was analyzed via elemental analysis, 1H NMR, SEM, BET analysis, TG-DTG/DSC, TG-FTIR, and FTIR.

The yield of humic substance was calculated as the weight of extracted humic substance per weight of hazelnut husk. The yield was 18.8%.

The total (humic + fulvic acid)content of the extracted humic substance in the hazelnut husk was 67.5%, according to TS 5869 ISO 5073.

### 2.2. Analysis

Elemental analysis was performed on a LECO CHNS-932 apparatus (LECO Corp., St. Joseph, MI, USA).1H NMR spectra were recorded on an advance 400 MHz Varian NMR spectrometer (Varian, Inc., Palo Alto, CA, USA). A Zeiss Sigma 300 SEM (Carl Zeiss Microscopy GmbH, Oberkochen, Germany) was used to determine the morphological structures of the samples. The surface area and pore distributions [Barrett-Joyner-Halenda (BJH) method] of the humic substance were measured using a Micromeritics Gemini 2.00 BET instrument (Micromeritics Instrument Corporation, Norcross, GA, USA).

For the TG-DTG/DSC analysis, a NETZSCH STA 409 PC Luxx TG analyzer (NETZSCH-Gerätebau GmbH, Selb, Germany) was used to better understand the origin of the humic matter and characterize the thermal decomposition.TG-DTG/DSC experiments were performed at 25–1000 °C, at heating rates of 2.5, 5, and 10 °Cmin–1,under air (90 mL min–1) conditions. TheTG-DTG data were utilized to determine the decomposition kinetics of the humic substance.

The TG-FTIR analysis, performed using a PerkinElmer Pyris STA 600 thermogravimetric analysis &spectrum 1 FTIR spectrometer (PerkinElmer, Inc., Waltham, MA, USA), was conducted to analyze the gases released during the decomposition of the humic substance.TG-FTIR analysis was performed at 25–1000 °C with a heating rate of 10 °Cmin–1in an air atmosphere. FTIR spectra of the gases transferred from the TG instrument were drawn in the range of 4000–400 cm–1. The TG-DTG/DSC and TG-FTIR analyses are rapid and accurate instrumental methods to provide the opportunity to understand the geochemistry and origin of the humic substance.IR spectra were drawn in the range of 4000–400 cm−1 using a PerkinElmer spectrum one FTIR spectrometer.

## 3. Results and discussion

### 3.1. Elemental analysis

The elemental analyses are given in Table 1.The H/C and O/C ratios may show the presence of a higher aromatic structure humic substance. The amount of oxygen may show the presence of functional groups on the humic substance surface, and the same can be also said about hydrogen. They are mainly carboxylic and phenolic groups, and it will be displayed in their respective bands in the FTIR spectrum.

### 3.2. 1H NMR analysis

**Figure 1:**
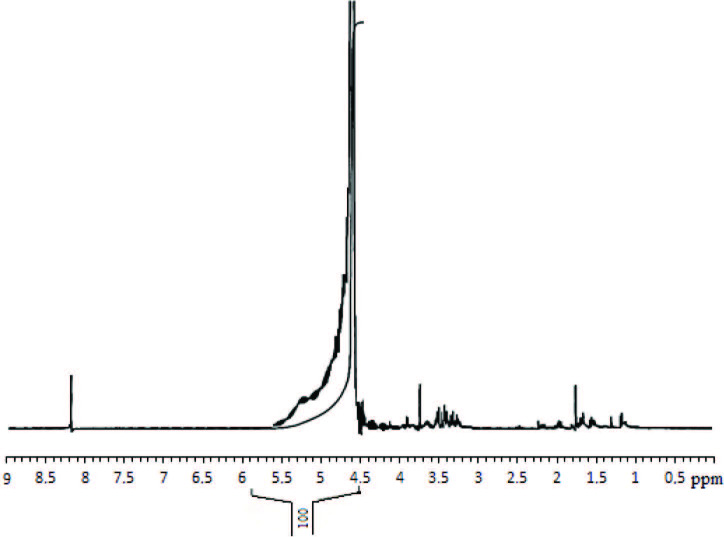
1H NMR (400 MHz, D2O) spectra of the humic substances.

1H NMR spectra (Figure 1) of the humic substance obtained from the hazelnut husks indicated 3 chemical regions: the aliphatic protons at 0.5–3 ppm, high proportions of the carbohydrate structures and heteroatom (especially oxygen atoms) content in the aliphatic groups at 3–4.2 ppm, and the aromatic resonance at 8–8.2 ppm. The spectra were agreement with the results in the literature and it has been stated that high proportions of aliphatic groups, carbohydrate structure, and aromatic molecules, which are important for the existence of the humic substances, were also found the humic substances obtained from hazelnut husks [29].

### 3.3. SEM analysis

**Figure 2:**
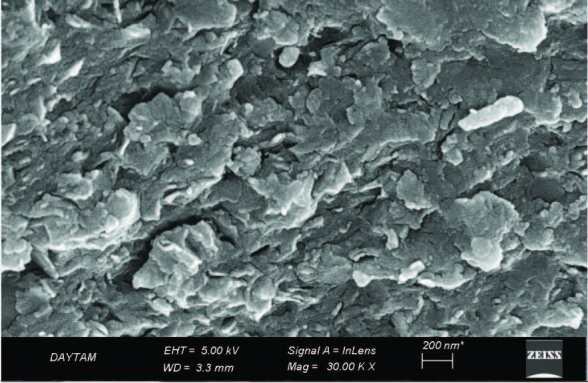

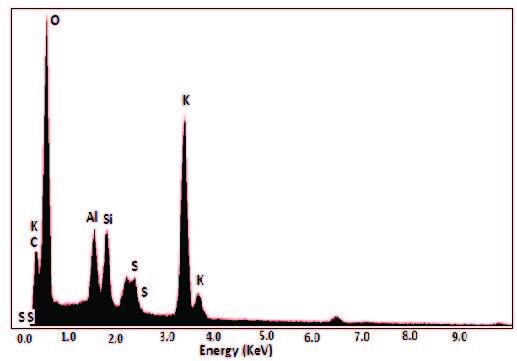
SEM image and EDS analysis of the humic substance from the hazelnut husks.

The SEM image indicated that the humic substance had compacted micro aggregates, as shown in Figure 2. Changlung et al. explained that the determination of the aggregate structure of humic substances is a very important function in the transportation of heavy metals in their surroundings [30]. In the literature, it was stated that information about the macro-molecular structure and shape of humic acid is necessary to evaluate the behavior of metal ions in humic acid-metal ion-mineral triple systems in the natural environment [31].

The elemental quantification of the humic substance was confirmed via energy-dispersive X-ray spectroscopy (EDS) analysis and shown in Figure 2. The EDS spectrum detected K, S, Al, Si, C, and O atoms.

### 3.4. BET analysis

**Figure 3:**
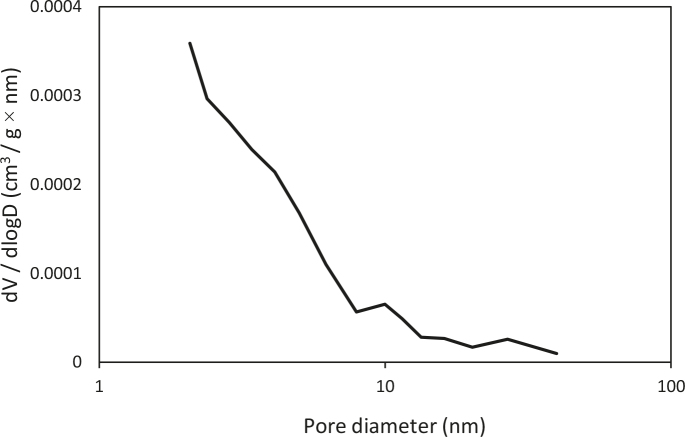
Pore distribution of the humic substance.

In Figure 3, the pore size distribution (PSD) of humic substance can be seen. The specific surface area (SBET) and pore distribution of the humic substance were examined. The BET surface area of the humic substance was SBET = 0.3214 m2/g. The BJH method (especially at the 2–50 nm pore diameter) is generally used to determine the PSD. The PSD of the humic substance indicated peaks in the micropore region (7.94–13.37 nm) and mesopore region (20.19–39.81 nm). In the literature, it has been emphasized that the BET surface areas of humic acid samples obtained from soil varied between 0.7 and 18 m2/g, and Dogan et al. reported the specific surface area of barium humate as 1.2 m2/g [12,32].

### 3.5. TG-DTG/DSC analysis

**Figure 4:**
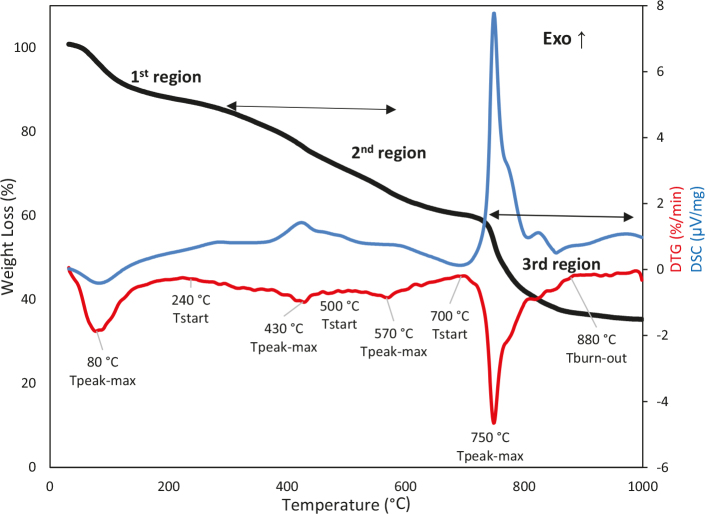
TG-DTG/DSC profiles of the humic substance.

Figure 4 represents the experimental results corresponding to the decomposition (combustion) of the humic substance inair atmosphere and at 10 °Cmin–1and a heating rate from 25 to 1000 °C. The TG-DTG/DSC curves indicated the mass losses and characteristic temperatures (reaction start temperature, maximum peak temperature, end temperature of the reaction). The characteristic temperatures were displayed on a DTG curve. It can be understood from the TG-DTG curves in Figure 4 that the oxidation of the humic substance had a 3-region decomposition graph under nonisothermal conditions. According to this:

Ø Region 1 (25–180 °C): The humic substance lost its moisture in this region. Weight loss was approximately 11.6%. The adsorbed waterin the layers or pores was initially removed at up to 180 °C. This situation appeared as a very sharp peak in the DTG graph at the same time as an endothermic peak in the DSC curve.

Ø Region 2 (180–700°C): In this region, the removal of the volatiles from the oxidation of the carbonaceous structures occurs in the humic substance. Weight loss was approximately 27.8% and there was a very small exothermic peak in the DSC curve. The small exothermic peak indicated that the burning had started.

Ø Region 3 (700–1000 °C): In this region, 32% mass loss was accompanied by a corresponding sharp exothermic peak in the DSC curve. At the end of decomposition, the residue was approximately 35.35% inorganic material according to the TG profile.

The literature comprises many studies about the decomposition of extracted humic matter from different materials (lignite, peat, soil, flooded soil, wastes, etc.) via TGA apparatus studies [8,33,34]. In these studies, it was observed that decomposition was complete at approximately 500–600 °C, but in the study of Oliviera et al., decomposition was not complete at 600°C, and a sharp exothermic peak was observed between 630 °C and 950 °C in the decomposition of a humic substance extracted with NaOH from Iara soils [8]. They explained that the mineral oxides or carbonates in organic matter decomposes in this region.

Dogan et al. produced barium humate from leonardite and examined it via TG analysis. They observed from the TG analysis that the barium humate had a higher ash content than the leonardite, and reported more inorganic materials like the humic substance that was obtained in the current study [12]. The decomposition temperature range of the barium humate from leonardite was between 600 °C and 850 °C. The results in the literature were very compatible with the results herein.

In the current study, the TG-DTG/DSC analysis was supported by the FTIR analysis of the humic substance (at 200, 500, 700, and 1000°C) and the TG-FTIR analysis; thus, it was possible to better explain the chemical structure of the extracted matter. It was sought toanswers questionslike which structures were formed and which gases were released.

### 3.6. TG-FTIR analysis

**Figure 5:**
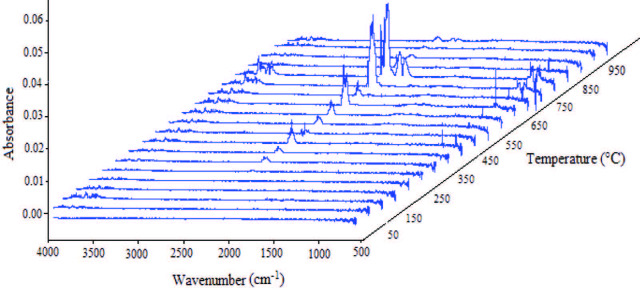
FTIR-3D graph of the gas products released from the decomposition of the humic substance at different temperatures.

TG-FTIR analysis, known as evolved gas analysis, was performed at room temperature to 1000 °C at a heating rate of 10 °C/min, and the FTIR spectra of the emitted gases are shown in Figure 5, in the range of 4000–500 cm–1.

The analysis was an appropriate approach that provided monitoring of the decay paths [19,35–37]. The O-H spectrum initially drew attention in the range of 4000–3600 cm–1in Figure 5. With the increase of temperature, the peak of CO2 in the range of 2400–2220 cm–1 at 350 °C began to appear and the peak increased at 450 °C. At 500 °C, the CO2 peak decreased, but at 650 °C, the peak value increased again. The CO2 peak decreased at 700 °C and reached its maximum value at 750–800 °C. In addition, the CO spectrum at 2220–2050 cm–1 appeared at 800 °C. At 4000–3600 cm–1, the O-H spectrum was at a maximum value at 750–800 °C. For the heating rate of 10 °C/min, there was a significant match between the initial temperatures specified, the maximum peak temperatures in the DTG profile, and the peak densities of the gases released in the TG-FTIR analysis. It was previously stated in the TG-DTG analysis that the decomposition process of the humic substance occurs in three steps. The initial and final temperatures of these regions were shown in the DTG curve. The amount of gases released in the TG-FTIR analysis had the highest value at maximum peak temperatures in the DTG curve. The most rapid decomposition occurred at the peak-max temperature in the DTG curve. The fact that the peak density (especially CO2) of the gases in the TG-FTIR spectrum was higher than the peak of other temperatures supported this situation.

Based on the TG-FTIR analysis, it can be said that CO2 intensity released from the exothermic reaction formed between 650 °C and 850 °Cwas stronger than the other temperatures, and the reaction in the range of the temperature consisted of a carbonaceous structure-decomposition.

Gas products formed by decomposition of the humic substance were monitored usingTGA-FTIR.

### 3.7. FTIR analysis

**Figure 6:**
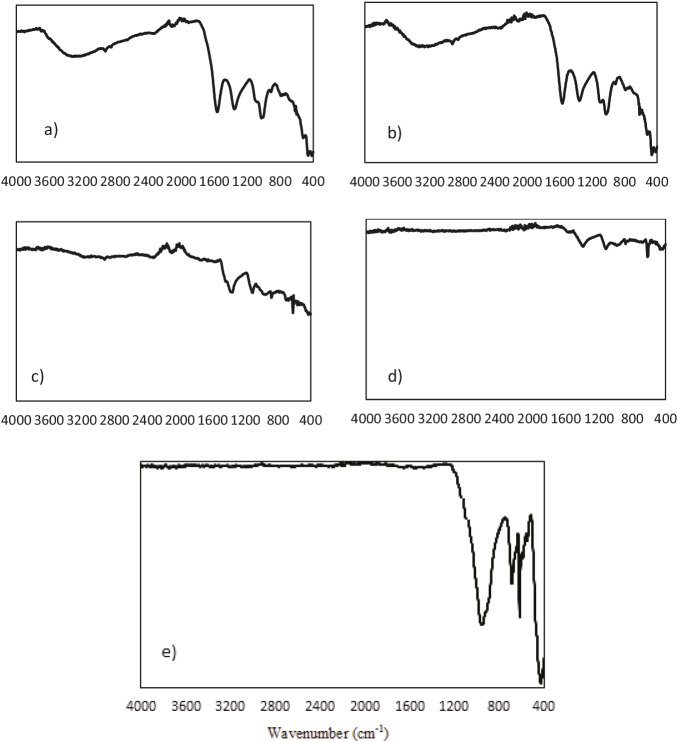
FTIR spectrum of the humic substance at a) original, b) 200 °C, c) 500 °C, d) 700 °C, and e)1000 °C.

FTIR analysis showed the changes in the functional groups and compositions in the humic substance during the decomposition process. The FTIR analysis, shown in Figure 6, was related to the experimental results of the samples at certain temperatures, i.e. 200, 500, 700, and 1000 °C. It is noteworthy that the generally observed species included O-H, C-H, aromatic C=C, and C-O, and C=O, Si-O, and Me-O/silicate-containing structures. Table 2 contains the list of FTIR bands and their functional groups. The results were in agreement with those in the literature [21,37].

**Table 2:** Major infrared bands of the humic substances from the hazelnut husks.

Wavenumber (cm-1)	Assignment
3700–3000	O-H stretching, N-H stretching
3000–2800	Aliphatic C-H stretching
1800–1450	C=O stretching of COOH and ketones, aromatic C=C, COO- symmetric stretching, C=N and C=O stretching
1450–1400	CO3–2 for common inorganic compounds
1400–1180	CH3 symmetric stretching
1180–1000	C-O-C stretching of polysaccharide or polysaccharide-like substances, and Si-O-C, -OH
˂1000	Me-O stretching

O-H stretching was observed at 3700–3000 cm−1, C-H aliphatic and aromatic stretching at 3000–2800 cm–1, C=C, C=O stretching of COOH and ketones at 1800–1450 cm–1, CO3–2 for common inorganic compounds at 1450–1400 cm–1, and CH3 symmetric stretching at 1400–1180 cm–1. Aliphatic ether C-O and alcohol C-O stretching and –OH stretching are usually observed at 1180–1000 cm–1.The stretching vibrations of the –COO, −CH, and −OH groups in the FTIR spectra indicates the rich oxygen-containing functional groups on the surface of the pure potassium humate.Me-Obands are usually seen below 1000 cm–1, which are caused by interatomic vibrations [37,38].

It was understood that the temperature rise caused the decreasing of some groups, and even the disappearance of some groups, such as O-H at 3700–3200 cm−1, C-H at 3000–2800 cm–1,and C=C, C=O at 1800–1500 cm–1. At 500 °C, no C=C or C=O peak appeared at 1800–1500 cm–1. Moreover, CO3–2 and CH3 groups at 1500–1200 cm–1, C-O stretching of polysaccharide or polysaccharide-like substances, Si-O of silicate impurities at 1200–1000 cm–1, and Me-O bands with peaks ˂1000 cm–1 continued to exist at 700 °C,but at 1500–1200 cm–1, the significant peak at 700 °C disappeared at 1000 °C. In fact, it was found that approximately 35.35% of the inorganic material (the residue) was determined via the TG profile. Considering the FTIR results together with TG data, it can be deduced that the peak at 1500–1200 cm–1 in the FTIR spectra caused a big exothermic peak in the TG profile.

The TG-FTIR spectrum in Figure 5 and FTIR spectrum in Figure 6 show that the carbonaceous structure of the humic substance from hazelnut husks via extraction with KOH was not damaged or impaired, even at 700 °C.

Humic substances may vary considerably in their inorganic components due to differences in the extraction and purification process. Generally, the ashes of a humic substance consist mainly of Si and Al components, comprising small amounts of alkaline/alkaline earth elements, Fe and Ti, and trace heavy metals [8]. In this study, the inorganic components of the humic substance via EDS analysis were previously determined and presented in Figure1.

### 3.8. Kinetics analysis

**Figure 7:**
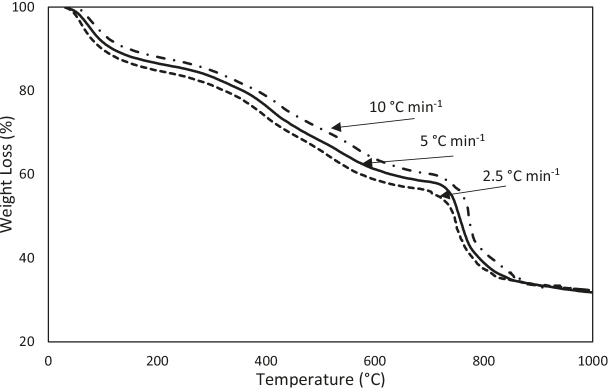
TG profiles of the humic substance at different heating rates.

**Figure 8:**
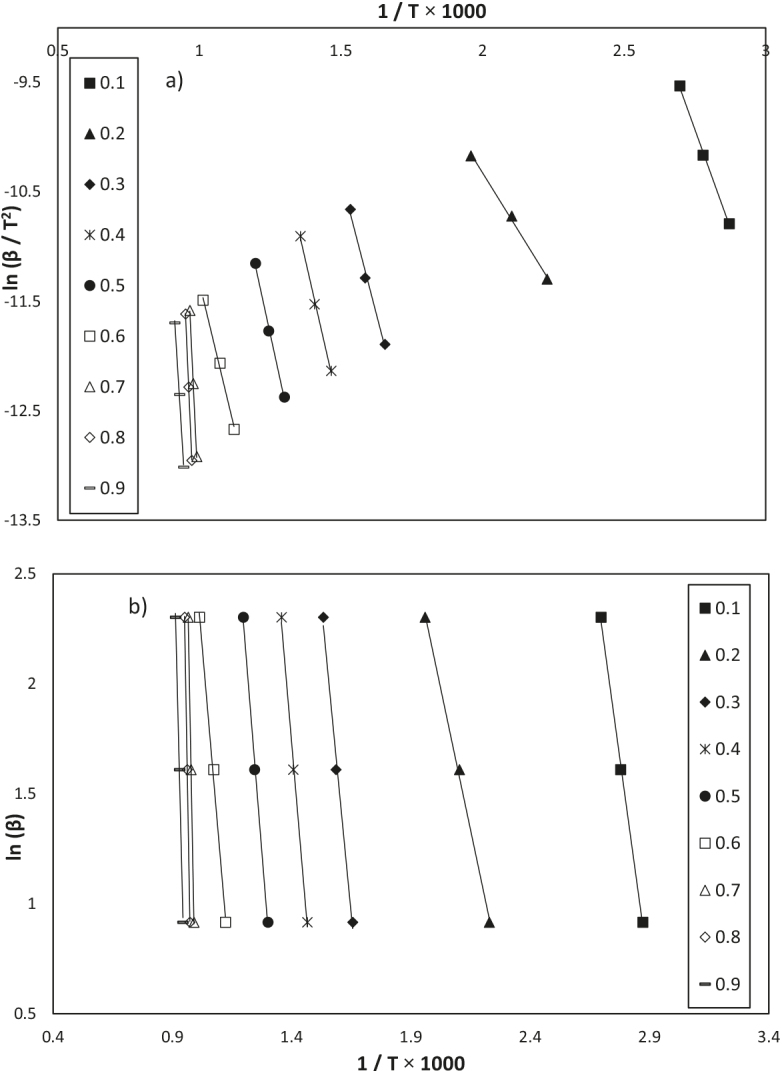
Curves fitting a) KAS method and b) FWO method in the conversion values (α) 0.1–0.9 and heating rates 2.5, 5, and 10 °Cmin–1.

The TG experiments on the humic substance were performed at different heating rates (2.5, 5, and 10 °Cmin–1), as shown in Figure 7. In the kinetics analysis, the overall decomposition process of the humic substance was assumed to occur from 25°C to 1000 °C. As can be seen in Figure 8, the kinetics analysis was performed using the KAS and FWO methods, and the activation energies for each conversion value were calculated (Table 3). In the KAS method, the activation energies were calculated from the slope of ln(β/T2) versus the (1 / T × 1000) graph, and in the FWO method, from slope of ln(β) versus the (1/T × 1000) graph. High correlation coefficient (r2) values were obtained.

**Table 3:** Activation energies (Ea) and correlation coefficients (r2) calculated using the KAS and FWO methods.

KAS method			FWO method	
α	Ea (kJ mol–1)	r2	Ea (kJ mol–1)	r2
0.1	59.8	0.9985	62.5	0.9987
0.2	37.8	0.987	40.6	0.9982
0.3	82.9	0.9915	88.8	0.9931
0.4	94.1	0,9964	100.6	0.997
0.5	99.9	0.9965	106.7	0.9971
0.6	89.4	0.9954	99.8	0.9969
0.7	460.8	0.9996	454.2	0.9996
0.8	495.1	0.9999	487.0	0.9999
0.9	347.3	0.9974	347.2	0.9977

It was seen that none of the linear fit plots were parallel to each other, but they were parallel in the conversion range of 0.1–0.2, 0.3–0.6, and 0.7–0.9, and the activation energies had low, medium, and high values in these ranges. For the conversion range of 0.1–0.2, the mean activation energy was approximately 48.8 kJ mol–1.This was a low activation energy value that corresponded to the removal of moisture inregion 1. For the conversion range of 0.3–0.6, the mean activation energy is 91.6 kJ mol–1. This was a medium activation energy value that corresponded to the removal of the volatiles from the oxidation of the carbonaceous structures in the humic substance in region 2. Aromatic carbonyl/carboxyl compounds decompose at up to 700 °C. The mean activation energy was 477.9 kJ mol–1 for the conversion rangeof 0.7–0.8, and 347.3 kJ mol–1 for the conversion valueof 0.9. In region 3, corresponding to these conversion ranges, CO3–2 for the common inorganic compounds and -CH3compounds were released from the humic substance in temperature range of 700–1000 °C.

In the literature, a kinetics analysis of the humic substance extracted from different soil samples in an air and nitrogen atmosphere were investigated using the FWO method [8]. In that study, the samples were characterized by comparison of the activation energies associated with their dehydration and thermal decomposition. In another study, a kinetics analysis of the humic substance in a nitrogen atmosphere was also investigated using the FWO method. The activation energy was calculated as 165.9 kJ mol–1[16].

## 4. Conclusion

In the current study, a humic substance was obtained from hazelnut husks. Various biological wastes and sources with high organic matter content,such as hazelnut husks,can be evaluated as a humic acid source; however, extensive analysis should be performed for this assessment. It is necessary to reveal similar and different features of the humic substances, because humic substances can be utilized in different areas. To investigate the decomposition of humic substance by thermal analysis (TG-DTG/DSC) offers the opportunity of understanding its origin and structure.

In this study, potassium humate was produced via alkali extraction, as a simple and cheap method for hazelnut husk, which is a waste organic matter. It was seen that the obtained humic substance had prominent characteristic properties. At high temperatures, it was seen that it differed, especially with regards to its thermal stability, behavior, and decomposition kinetics,when compared to the data in the literature. A significant part of the humic substance decomposed at above 700 °C, and had an activation energy of approximately 477.9 kJ mol–1. During the decomposition process, data corresponding to the kinetic analysis of a humic substance may also provideinformation about other high-temperature decomposition/combustion processes.

Thermal analysis experiments with FTIR analysis indicated that the humic substance decomposed at above 700 °C, and the inorganic components made the carbonaceous structure very resistant. In the other words, the components of the humic substance attained high thermal stability. The results could also be verified by the high activation energies (in region 3) for the decomposition in an air atmosphere. As a result, it can be said that a humic substance with high thermal stability can be obtained from waste material (hazelnut husk).

**Table:** Abbreviation list

β heating rate	(°C min–1)
Ea activation energy	(kJ mol–1)
R gas constant	(kJ mol–1 K–1)
A preexponential factor	
f(α) reaction mechanism	
g(α) integral function of f(α)	
